# Altered expressions of CXCR4 and CD26 on T-helper lymphocytes in hereditary hemorrhagic telangiectasia

**DOI:** 10.1186/s13023-021-02139-y

**Published:** 2021-12-14

**Authors:** Alexandre Guilhem, Pierre Portalès, Sophie Dupuis-Girod, Sophie Rivière, Thierry Vincent

**Affiliations:** 1 CHU de Montpellier, Médecine interne et maladies multi-organiques de l’adulte, Hôpital Saint Eloi, Montpellier, France; 2 CHU de Montpellier, Laboratoire d’immunologie, Hôpital Saint Eloi, Montpellier, France; 3 Centre National de référence Maladie de Rendu-Osler, Service de génétique Hôpital Mère-Enfant, Hospices Civils de Lyon, Bron, France

**Keywords:** Hereditary hemorrhagic telangiectasia, T-helper lymphocytes, CXCR4, CD26, Susceptibility to infection

## Abstract

**Background:**

Hereditary hemorrhagic telangiectasia (HHT) is a rare genetic disease characterized by a deregulated neo-angiogenesis. Besides a mainly vascular phenotype (muco-cutaneous telangiectases, arteriovenous malformations), a specific risk of infection is suggested by case series of severe and atypical infections as well as by reports of decreased T and natural killer (NK) lymphocyte counts. As some evidence supports a dysregulation of the CXCR4/CXCL12 chemotactic axis of HHT endothelial cells, we hypothesized that a similar phenomenon could occur on lymphocytes.

**Methods:**

Eighteen HHT patients with history of severe infection (HSI) were matched in age and sex with 18 HHT without HSI and 18 healthy control subjects (HC). We assessed the cell count and the surface expression of CXCR4 and CD26 (CXCL12 inactivating peptidase) of circulating T-helper and T-cytotoxic lymphocytes (including naive, memory and activated subsets) and NK cells.

**Results:**

The overall HHT group of 36 patients exhibited a reduction of circulating T-helper lymphocytes compared to HC (median: 517 vs. 1026 cells/mm^3^, *p* < 0.0001), correlated with age (r =  − 0.46, *p* = 0.005), requirement of intravenous iron or blood transfusions (median: 291 vs. 627 cells/mm^3^, *p* = 0.03) and CXCR4 surface expression (r = 0.353, *p* = 0.0345). CXCR4 and CD26 membrane expression were both decreased on HHT T-helper lymphocytes (median MFI ratio: 4.49 vs. 5.74 for CXCR4 and 3.21 vs. 4.33 for CD26, *p* = 0.03 and 0.0018 respectively) with an unchanged CXCR4/CD26 ratio. The HHT group with HSI had a higher CXCR4/CD26 ratio on the total T-lymphocyte population, as well as on the T-helper population and its naive subset (median on naive T-helper cells: 2.34 vs. 1.32, *p* = 0.0002).

**Conclusions:**

Our findings support a dysregulation of the CXCL12/CXCR4 chemotaxis of T-helper lymphocytes in HHT patients, potentially linked to their T-helper lymphopenia and susceptibility to infection.

**Supplementary Information:**

The online version contains supplementary material available at 10.1186/s13023-021-02139-y.

## Background

Hereditary hemorrhagic telangiectasia (HHT) is a vascular genetic disease with an estimated prevalence of 1 in 5000–8000. Mutations in several genes of the BMP9/BMP10 signaling pathway (ENG and ACVRL1 mainly) result in a deregulated neo-angiogenesis. The inheritance pattern is autosomal dominant, and the penetrance is usually complete after 50 years. The clinical presentation includes spontaneous and recurrent epistaxis (frequently complicated by iron-deficiency anemia), muco-cutaneous telangiectases (face, oral cavity, hands), and less frequently visceral arteriovenous malformations (AVM) of the lungs, the liver, the digestive tract or the central nervous system [1].

Besides this vascular phenotype, some studies reported atypical and severe infections, including cerebral abscesses and musculoskeletal infections, affecting up to 15% of the patients. The actual prevalence and clinical significance of these manifestations have yet to be confirmed by large prospective studies comparing this risk with that of the general population. The most specific events are cerebral abscesses with anaerobic bacteria, generally attributed to septic emboli allowed by right-to-left cardiac shunting due to pulmonary AVM. Patients also seem to be concerned more frequently by musculoskeletal infections involving *Staphylococcus aureus*, which may be associated with bacteremia provoked by prolonged nose packing or/and lesions of the nasal mucosa [2, 3]. In addition to these mechanical factors, innate immunity is suspected to be altered in HHT: functional deficits of neutrophils and monocytes/macrophages (phagocytosis, oxidative burst, NETs formation) have been reported in humans [4, 5] and mice [6], but their exact clinical meaningfulness as well as their underlying mechanism are still to be elucidated. Intriguingly, immunological studies have highlighted a T and NK lymphopenia predominant on naive T-helper cells [5, 7]. Although very low lymphocyte counts were observed in some patients, no clear association with infectious risk has been established to date.

The chemokine CXCL12 (formerly named SDF1) and its cellular receptor CXCR4 constitute a powerful chemotactic axis for many cell types. It is in particular used by T lymphocytes for trafficking and homeostasis [8]. CD26 is a known CXCL12 inactivating peptidase, functionally associated with CXCR4 on T lymphocytes [9]. Besides this enzymatic activity, CD26 acts as a co-stimulatory molecule during T-lymphocyte activation [10, 11]. In endothelial cells, CXCL12 and CXCR4 expressions are regulated by BMP9 in an endoglin-dependent manner: BMP9 and hypoxia are additive inducers of CXCL12 release while surface CXCR4 is downregulated [12]. Imbalance of this chemotactic axis has also been described on peripheral blood mononuclear cells (MNCs) in one study. The migration capacities of HHT MNCs towards a CXCL12 gradient were reduced as a result of a CD26 hyper-expression, which was overcoming elevated surface levels of CXCR4 [13].

Here, we describe the CXCR4 and CD26 expression levels in different lymphocyte subsets of HHT patients with history of severe infection (HSI), in comparison with HHT patients without HSI and with healthy control subjects (HC) matched in age and sex. We provide several elements supporting an alteration in the CXCL12/CXCR4 axis on T-helper lymphocytes in HHT patients, possibly related to their specific infectious risk and T-helper lymphopenia.

## Results

### Demographics and clinical characteristics

**Fig. 1:**
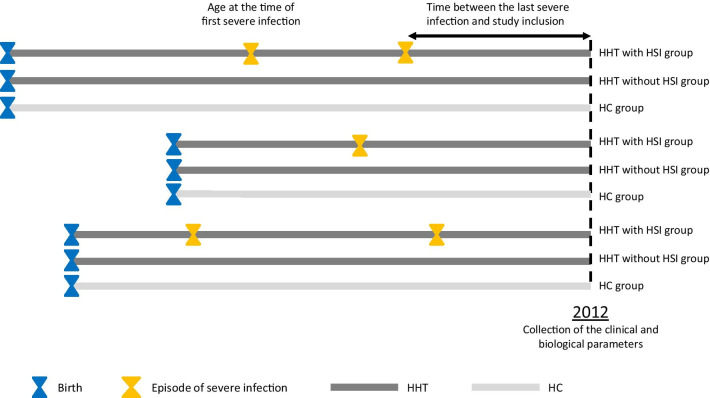
Schematic timeline of the study. HC: health control, HSI: history of severe infection

According to the schematic timeline shown in Fig. 1, 18 HHT patients with at least one history of severe infection were enrolled between January and October 2012, concomitantly with 18 HHT patients without any history of severe infection and with 18 HC. The main characteristics are detailed in Table 1. With a median age of 60 years and a male predominance, the HHT group was well-balanced in term of causative mutation and epistaxis intensity during the last month before inclusion. One third of the group had at least one pulmonary AVM. A majority of patients (56%) required intravenous (IV) treatments for iron-deficient anemia (IV iron or blood transfusion), and 4 (11%) were under bevacizumab.

**Table 1:** Demographics and clinical characteristics of the subjects

	HHT (n = 36)	Controls (n = 18)	
Age (mean, SD)	60.2 (10.4)	59.7 (10.2)	*p* = 0.868
Sex (males, %)	26 (72%)	13 (72%)	*p* = 1
History of severe infection (n, %)	18 (50%)	0 (0%)	
Mutation (n, %):			
ENG	18 (50%)		
ACVRL1	16 (44%)		
SMAD4	0 (0%)		
Not identified	2 (6%)		
Visible telangiectases (median, min–max)	70 (0–242)		
Epistaxis frequency^1^ (n, %)			
Daily	13 (36%)		
Weekly	13 (36%)		
Monthly or more rarely	10 (28%)		
Arteriovenous malformations (n, %^2^)			
Lung^3^	12 (34%)		
Digestive tract	19 (73%)		
Liver	15 (44%)		
Brain	6 (25%)		
Treatment (n, %)			
IV iron or blood transfusion (at least 5)	20 (56%)		
Tranexamic acid	6 (17%)		
Bevacizumab	4 (11%)		

n: Number of patients

^1^During the last month before blood sampling

^2^% of patients with a screening performed for the specified organ

^3^Only those large enough to be considered for treatment (usually diameter of the feeding artery > 3 mm)

Thirty episodes of severe infection were identified in the medical history of 18 HHT patients (1.67 episodes/patient): 7 cerebral abscesses, 8 osteoarticular infections (including 6 cases of spondylodiscitis), 5 cutaneous or soft tissue infections, 3 cases of bacteremia, 2 cases of pneumonia (including one with pulmonary abscess), 2 cases of pyelonephritis, 1 case of prostatitis, 1 case of sigmoiditis and 1 case of bartholinitis. The infectious agents were: *Staphylococcus aureus* in 10 cases, anaerobic bacteria from the oral cavity in 9 cases, 4 cases of *Enterobacteriaceae*, and 2 anaerobic bacteria from the digestive tract (no microbe identified in 5 cases). The median age at the first episode of infection was 46 years (min–max: 21–67 years). The median duration between the last episode of infection and the inclusion in the study was 2.3 years (min–max: 1–16 years). Two episodes were classified as septic shock and one as sepsis according to the Sepsis-3 consensus definitions [14].

### Surface expression of CXCR4 and CD26 on T and NK lymphocytes

**Fig. 2:**
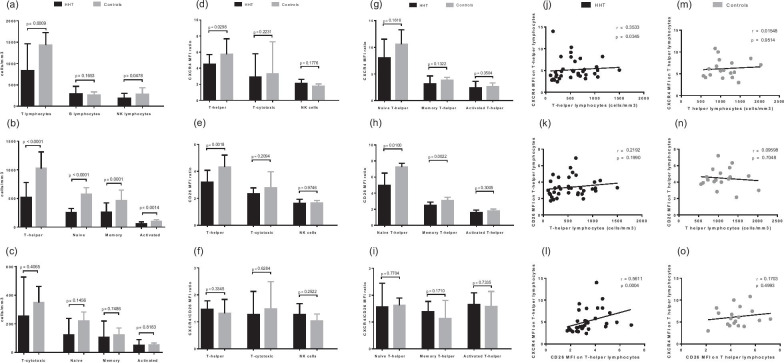
Lymphocyte subpopulations and CXCR4-CD26 surface expressions. In 36 HHT patients (black bar or dots) and 18 healthy control subjects (grey bar or dots) matched by age and sex: **a** absolute counts of T, B and NK lymphocytes, **b** absolute counts of T-helper lymphocytes (total, naive, memory and activated), **c** absolute counts of T-cytotoxic lymphocytes (total, naive, memory and activated), **d** CXCR4 expression on T-helper, T-cytotoxic and NK lymphocytes, **e** CD26 expression on T-helper, T-cytotoxic and NK lymphocytes, **f** ratio of CXCR4/CD26 expressions on T-helper, T-cytotoxic and NK lymphocytes, **g** CXCR4 expression on naive, memory and activated T-helper lymphocytes, **h** CD26 expression on naive, memory and activated T-helper lymphocytes, **i** ratio of CXCR4/CD26 expressions on naive, memory and activated T-helper lymphocytes, **j** correlation between absolute counts of T-helper lymphocytes and their expression of CXCR4 in the HHT group, **k** correlation between absolute counts of T-helper lymphocytes and their expression of CD26 in the HHT group, **l** correlation between CXCR4 and CD26 surface expression on T-helper lymphocytes in the HHT group, **m** correlation between absolute counts of T-helper lymphocytes and their expression of CXCR4 in the control group, **n** correlation between absolute counts of T-helper lymphocytes and their expression of CD26 in the control group, **o** correlation between CXCR4 and CD26 surface expression on T-helper lymphocytes in the control group. MFI: mean fluorescence intensity. Results are presented in bar charts with median and interquartile ranges (for comparisons) or individual values with Spearman r coefficients (for correlations). Comparisons between groups were performed using the Mann–Whitney *U*-test. Correlations between variables were performed using the Spearman’s rank test. *P*-values are shown

By comparing all the 36 HHT patients (with or without HSI) to the 18 HC in Fig. 2, we observed a significant decrease in total T-lymphocyte count in the HHT group (median: 833 vs. 1436 cells/mm^3^, *p* = 0.0009), linked to a reduction of the T-helper sub-population (median: 517 vs. 1026 cells/mm^3^, *p* < 0.0001) with a more pronounced impact on the naive cells (median: 254 vs. 577 cells/mm^3^, *p* < 0.0001). The NK lymphocytes were equally diminished (median: 184 vs. 284 cells/mm^3^, *p* = 0.048).

CXCR4 and CD26 had both a lower mean fluorescence intensity (MFI) on T-helper lymphocytes in the HHT group (median MFI ratio: 4.49 vs. 5.74 for CXCR4 and 3.21 vs. 4.33 for CD26, *p* = 0.03 and 0.0018 respectively). Decreased membrane expression was also significant for CD26 on naive and memory subsets (median MFI ratio: 4.96 vs. 7.26 for naive and 2.48 vs. 3.08 for memory, *p* = 0.01 and 0.0022 respectively). T-cytotoxic lymphocytes and their subsets exhibited no significant difference in CXCR4 and CD26 MFI ratio (data not shown). The CXCR4/CD26 ratio was not altered in the NK and the different T lymphocyte subsets.

A weak but significant positive correlation was observed in the HHT group between the surface expression of CXCR4 on T-helper lymphocytes and their absolute count (r = 0.353, *p* = 0.0345). A stronger positive correlation was present between the CXCR4 and the CD26 expression on this subset (r = 0.561, *p* = 0.0004). These two observations were not found in the HC group.

**Fig. 3:**
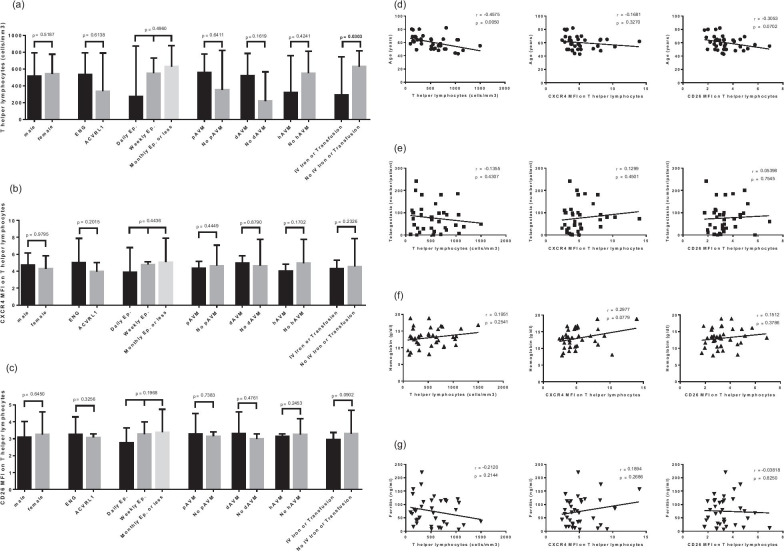
Influence of main HHT characteristics on T-helper count and their CXCR4 and CD26 surface expressions. Among the 36 subjects of the HHT group, comparisons of the T-helper count (**a**), the CXCR4 MFI ratio (**b**) and the CD26 MFI ratio (**c**) according to sex (male: 26, female: 10), mutations (ENG: 18, ACVRL1: 16), frequency of epistaxis (daily: 13, weekly: 13, month or less: 10), pulmonary AVM (with: 12, without: 23), digestive AVM (with: 19, without: 7), hepatic AVM (with: 15, without: 19), IV iron or transfusion (with: 20, without: 16). Correlations between T-helper count, CXCR4 expression, CD26 expression and age (**d**), number of telangiectases per patient (**e**), hemoglobin (**f**), ferritin (**g**). MFI: mean fluorescence intensity, Ep.: epistaxis, pAVM: pulmonary arteriovenous malformation, dAVM: digestive arteriovenous malformation, hAVM: hepatic arteriovenous malformation. Results are presented in bar charts with median and interquartile ranges (for comparisons) or individual values with Spearman r coefficient (for correlations). Comparisons between groups were performed using the Kruskal–Wallis or the Mann–Whitney *U*-test. Correlations between variables were performed using the Spearman’s rank test. *P*-values are shown

The influence of the main HHT characteristics on T-helper count, the CXCR4 MFI ratio and the CD26 MFI ratio was tested in Fig. 3. Patients receiving IV iron or blood transfusions showed a significant reduction of T-helper count (median: 291 vs. 627 cells/mm^3^, *p* = 0.03). The T-helper count was also correlated with the age of the patients (r =  − 0.46, *p* = 0.005). The decrease of the CD26 MFI ratio followed the same tendencies for age and IV treatment of anemia, without reaching the statistical threshold. Another positive tendency was found between CXCR4 MFI ratio and hemoglobin level. We did not observe any differences between ENG and AVCRL1-mutated patients.

A multivariate analysis was carried out to assess the independence of the correlations between T-helper absolute count, age, requirement of IV treatment for iron-deficiency anemia and CXCR4 MFI ratio on T-helper cells, in the HHT group. The results of the multivariable linear regression are shown in Additional file 1: Table S1. Only the age of the HHT patients remained significantly related to the T-helper cell count (*p* = 0.042).

### Clinical and biological parameters associated with history of severe infection

By comparing the clinical characteristics of the 18 HHT patients with HSI to the 18 without HSI (Table 2), we observed a higher frequency of pulmonary AVM (50% vs. 18%, *p* = 0.044). The proportions of ENG and ACVRL1 mutated patients were not different between groups. The comparison of the immunological parameters revealed a higher expression of CXCR4 on naive T-helper lymphocytes (median MFI ratio: 9.65 vs. 7.01, *p* = 0.03) and a lower expression of CD26 on naive T-helper lymphocytes (median MFI ratio: 4.59 vs. 5.73, *p* = 0.03) in the HSI group. Consequently the CXCR4/CD26 ratio of this subset was higher in the HSI group (median: 2.34 vs. 1.32, *p* = 0.0002). Similarly, we observed in the HSI group an increase of the CXCR4/CD26 ratio on the T-helper (median: 1.58 vs. 1.37, *p* = 0.01) and the total T lymphocyte populations (median: 1.63 vs. 1.32, *p* = 0.04). This ratio was not significantly different between patients with and without pulmonary AVM (median: 2.15 vs. 1.51, *p* = 0.71).

**Table 2:** Comparisons of clinical and biological characteristics (a) and lymphocyte parameters (b) between HHT patients with or without history of severe infection

	HHT with HSI	HHT without HSI	*p*
(a) Clinical and biological characteristics			
Age (mean, SD)	60.0 (10.3)	60.4 (10.5)	0.91
Sex (male, %)	13 (72%)	13(72%)	1
Mutation (n, %)^1^			
- ENG	11 (61%)	7 (39%)	0.49
- ACVRL1	7 (39%)	9 (50%)	
Epistaxis frequency (n, %)			
- Daily	6 (34%)	7 (39%)	0.56
- Weekly	8 (44%)	5 (28%)	
- Monthly or more rarely	4 (22%)	6 (34%)	
Visible telangiectases (median, min–max)	58 (0–242)	90 (0–200)	0.39
Arteriovenous malformations (n, %^2^)			
- Lung^3^	**9 (50%)**	**3 (18%)**	**0.044**
- Digestive tract	7 (63%)	12 (80%)	0.35
- Liver	8 (44%)	7 (44%)	0.97
- Brain	5 (29%)	1 (14%)	0.78
Treatment (n, %)			
- IV iron or blood transfusion (at least 5)	8 (44%)	12 (67%)	0.14
- Tranexamic acid	2 (11%)	4 (22%)	0.37
- Bevacizumab	1 (6%)	3 (17%)	0.29
Basic biological parameters (median, min–max):			
Hemoglobin (g/l)	135 (80–188)	126 (78–157)	0.16
Platelets (G/l)	245 (178–388)	262 (236–488)	0.15
Leucocytes (G/l)	5.44 (3.00–8.92)	5.23 (3.02–10.23)	0.95
Neutrophils (G/l)	4.06 (1.73–7.65)	4.02 (1.94–8.19)	0.67
Lymphocytes (G/l)	0.92 (0.3–2.4)	1.02 (0.4–2.34)	0.89
C reactive protein (mg/l)	2.63 (0.2–8.4)	1.85 (0.2–7.3)	0.63
Ferritin (ng/ml)	76 (9–158)	57 (7–221)	0.43
Creatinine clearance (MDRD, ml/min)	85 (36–107)	86.5 (42–140)	0.52
Immunoglobulin G (g/l)	8,73 (2.7–16.8)	9.16 (3.16–16.9)	0.87
Immunoglobulin A (g/l)	2.10 (1.04–4.3)	2.23 (1.07–4.06)	0.64
Immunoglobulin M (g/l)	0.69 (0.36–1.79)	0.72 (0.19–1.9)	0.97
(b) Lymphocyte parameters (median, min–max)			
T lymphocytes (cells/mm3)	841 (219–2116)	829 (214–1831)	0.44
T-helper lymphocytes (cells/mm3)	517 (96–1499)	550 (123–1061)	0.83
T-cytotoxic lymphocytes (cells/mm3)	339 (90–848)	187 (51–1110)	0.12
NK lymphocytes (cells/mm3)	211 (12–989)	170 (55–946)	0.64
Naive T-helper lymphocytes (cells/mm3)	273 (8–978)	201 (14–630)	0.55
Memory T-helper lymphocytes (cells/mm3)	236 (79–519)	280 (109–798)	0.60
Activated T-helper lymphocytes (cells/mm3)	59 (19–151)	58 (30–215)	0.78
CXCR4 MFI on T lymphocytes	3,87 (2.17–18.36)	3.74 (2.20–14.30)	0.80
CXCR4 MFI on T-helper lymphocytes	4.80 (2.91–14.00)	4.17 (2.40–8.20)	0.48
CXCR4 MFI on Naive T-helper lymphocytes	**9.65 (4.48–35.00)**	**7.01 (3.34–14.20)**	**0.03**
CXCR4 MFI on memory T-helper lymphocytes	3.06 (2.28–9.79)	3.33 (1.91–6.00)	0.64
CXCR4 MFI on activated T-helper lymphocytes	2.40 (1.71–8.15)	2.49 (1.63–4.00)	0.97
CD26 MFI on T lymphocytes	2.71 (1.41–5.13)	2.90 (2.21–6.54)	0.13
CD26 MFI on T-helper lymphocytes	3.01 (1.72–5.75)	3.26 (2.17–6.92)	0.19
CD26 MFI on naive T-helper lymphocytes	**4.59 (1.65–9.33)**	**5.73 (4.17–14.1)**	**0.03**
CD26 MFI on memory T-helper lymphocytes	2.27 (1.58–3.48)	2.58 (2.00–5.08)	0.09
CD26 MFI on activated T-helper lymphocytes	1.63 (1.32–2.95)	1.57 (1.13–2.77)	0.29
CXCR4/CD26 ratio on T lymphocytes	**1.63 (0.52–5.45)**	**1.32 (0.45–4.38)**	**0.04**
CXCR4/CD26 ratio on T-helper lymphocytes	**1.58 (0.86–3.41)**	**1.37 (0.62–2.91)**	**0.01**
CXCR4/CD26 ratio on naive T-helper lymphocytes	**2.34 (0.87–5.68)**	**1.32 (0.51–3.01)**	**0.0002**
CXCR4/CD26 ratio on memory T-helper lymphocytes	1.38 (0.79–2.82)	1.39 (0.71–2.50)	0.25
CXCR4/CD26 ratio on activated T-helper lymphocytes	1.70 (0.70–2.96)	1.63 (0.99–2.65)	0.93

Statistically significant parameters are in bold

n: Number, HSI: History of severe infection, MFI: Mean fluorescence intensity

^1^No mutation identified for 2 patients, both in the HHT without HSI group

^2^% of patients with a screening performed for the specified organ

^3^Only those large enough to be considered for treatment (diameter of the feeding artery > 3 mm)

## Discussion

CXCR4 is the G protein-coupled receptor of the pleiotropic chemokine CXCL12. This chemotactic axis is fundamental during organogenesis, angiogenesis and homeostasis of the hematopoietic and immune systems. CXCL12 is highly expressed in bone marrow and is locally upregulated via HIF-1 after tissue injury to recruit stem cells and progenitors for damage repair [15, 16]. On T lymphocytes, CXCR4 is co-localized with CD26 (dipeptidylpeptidase IV), and both proteins are co-internalized after activation by CXCL12. They form a functional unit participating in chemotaxis towards bone marrow and lymph nodes [9]. This pathway is involved in several diseases, including cancer [17], rheumatoid arthritis [18] and HIV [19]. Here, we report for the first time elements supporting a dysregulation of this axis on T lymphocytes in HHT.

As already published [5, 7], HHT is associated with a T and NK lymphopenia despite the absence of any comorbidities or treatment known to cause lymphopenia [20]. The decrease of the T-helpers could be a consequence of a dysfunctional CXCL12/CXCR4 axis since we observed a weak but significant correlation between their CXCR4 surface expression and their absolute counts in the blood. This hypothesis is reinforced by the absence of such correlation in the control group. This phenomenon could be due to a direct effect of CXCL12 on naive T lymphocyte survival [21] or through disturbance in the T lymphocyte recirculation in lymph nodes and bone marrow, in which the CXCR4/CXCL12 axis is known to be important [8, 22]. The human aging of the immune system seems associated with a deregulation of CXCR4 expression on T-helper cell surface [23], but a such phenomenon is not observed in our study. The association between iron treatment and T-helper lymphopenia is more difficult to explain. It could be due to a direct effect of the treatment, maybe by increasing the oxygen reactive species in peripheral blood [24]. It also could be a consequence of the iron deficiency, which is known to induce a reduction in peripheral T cells and atrophy of the thymus [25]. As a third hypothesis, there could exist a confounding factor, such as a subpopulation of patients with a more strongly activated neo-angiogenesis, impacting on both iron requirements and lymphocyte recruitment. Interventional studies are necessary to differentiate these hypotheses, especially since a link between the risk of cerebral abscess and IV iron loading has been reported [26].

We observed a correlated decrease in CXCR4 and CD26 expressions on T-helper lymphocytes of HHT patients without modification of the CXCR4/CD26 ratio. This is in apparent contradiction with a previous report of an over-expression of CXCR4 and CD26 on mononuclear cells with a decreased CXCR4/CD26 ratio associated with chemotactic impairment [13]. This discrepancy could be due to a different pattern of CXCR4 and CD26 surface expression between lymphocytes and monocytes. The monocytes have never been specifically studied in human HHT but are known to express these markers [27, 28]. Moreover, the previous study included only ENG-mutated patients. On T-helper lymphocytes, CXCR4 under-expression seems to be a central phenomenon in the idiopathic CD4 lymphopenia [29], but the infectious profiles are quite different. An alternative explanation could be a chronic elevation of the CXCL12 plasma level, described in severe infection [30, 31] and recently in HHT [32]. Such an elevation is known to lead to continuous CXCR4 and CD26 internalization as a desensitization process [9, 33]. The under-expression of CXCR4 on T-helper lymphocytes could delay and mitigate their antigenic response by limiting their migration and activation capacities [34–36]. The decrease of CXCR4 and CD26 surface expression seems to spare the T-cytotoxic and the NK lymphocytes. This observation is surprising since both molecules have documented biological roles on these cell types [11, 35, 37–39]. It could result from their lower basal expression levels, limiting our ability to distinguish small variations.

We describe for the first time a significantly higher CXCR4/CD26 ratio on naive T-helper lymphocytes of HHT patients that have experienced at least one serious infectious event. The magnitude of this effect on clinical risk will require confirmation by further studies and may not be as large as the effect of pulmonary AVMs, the risk factor well described in HHT [2] and confirmed here. Since CXCR4 and CD26 are functionally linked, it is logical to suspect a higher chemotaxis of naive T-helper lymphocytes in HHT patients with HSI. This can appear paradoxical in an immunodeficient state, but such a phenomenon, extended to the whole immune system, has a central role in the physiopathology of the WHIM syndrome [40]. In this disease, most of the infectious phenotype seems to be due to the chemotactic dysfunction of the myeloid lineage, but patients also exhibit a decrease of NK and naive T lymphocytes [41]. However, the validity of a similar paradoxical mechanism underlying HHT-related infection profile is not obvious, since WHIM syndrome is due to a CXCR4 gain-of-function mutation, resulting in prolonged retention of leukocytes in bone marrow and, consequently, panleukopenia, whereas no evidence of such behaviour is currently available for naive T-helper lymphocytes in HHT patients. Of note, the CD26 expression on T lymphocytes is regulated upon activation in inflammatory contexts such as rheumatoid arthritis or allograft rejection [11, 42]. Therefore, inflammation could also participate in the shift of the CXCR4/26 ratio in HHT patients with a history of severe infection, despite the significant time elapsed between the last episode of infection and the time of enrollment.

Our study suffered from several limitations. The first is the low number of subjects, limiting the statistical analyses. This fact is inherent to studies on rare diseases. Nevertheless, the ability to identify pulmonary AVM as an infectious risk in HHT allows us to think that our population size was large enough to spot clinically relevant parameters. Another limitation comes from the absence of data about the lymphocyte profiles prior to the infectious episodes. We do not provide any proof that the modified expressions of CXCR4 and CD26 are stable over time, knowing that lymphocyte profiles have circadian and seasonal variations [43–45]. Nevertheless, the major lymphocyte subsets of a given individual have been reported with a low level of variability over time [46–48]. The assessment of CXCR4 and CD26 expression is only based on flow-cytometry data and should be confirmed by other techniques (RNA quantification, for example). Plasma levels of chemotactic agents (especially CXCL12) and functional assessment of T lymphocyte chemotaxis could have brought more interpretability in our results. These investigations should be conducted in future studies on this topic, as well as a more detailed evaluation of the Th1, Th2 and Th17 lymphocyte sub-populations.

## Conclusions

We report several findings suggesting that HHT is associated with an alteration of the CXCL12/CXCR4 chemotactic axis of T-helper lymphocytes. Patients exhibit a decrease of their total count in the blood correlated with a decrease of CXCR4 MFI ratio, a decrease of CXCR4 and CD26 surface expressions and a higher CXCR4/CD26 ratio on the naive subset in the group with a history of severe infection. As the CXCL12/CXCR4 chemotactic axis is important in many parts of the immune system, it may contribute to the greater susceptibility to infection observed in HHT.

## Methods

### Selection of participants and collection of baseline characteristics

Patients and HC were recruited between January and October 2012 in two French HHT centers (Montpellier and Lyon). Subjects were excluded if they were under 18 years of age or had any active or recent (< 3-month) conditions known to alter the immune system (infection, surgery, pregnancy, solid cancer or lymphoma, autoimmune disease, immunosuppressant or systemic corticosteroid therapy).

All patients included in the study fulfilled 3 or 4 of the Curaçao criteria and had undergone genetic analysis. A thoracic computed tomography scan and an echographic liver assessment were systematically proposed to all patients, according to the French guidelines for HHT diagnosis and treatment. Cerebral or gastrointestinal tract investigations were proposed only according to the clinical context, including symptoms, clinical signs and personal or familial histories.

Patients with a scheduled routine visit in the HHT centers were screened for inclusion during the medical consultation. They were considered to have a HSI if the infectious episode required at least two days of hospitalization (appendicitis excluded). For each patient included with at least one HSI, a HHT patient without any HSI and a HC were included, respecting a matching in sex and age (± 2 years).

Data regarding HHT symptoms, complete medical history, treatments and clinical status at the time of the study were collected during the consultation. Concerning pulmonary AVM, only those large enough to be considered for treatment were taken into account (usually with a diameter of the feeding artery > 3 mm). Blood samples were collected after the medical assessment. Standard biological measurements (blood cell count, C-reactive protein, ferritin, creatinine, immunoglobulins G-A-M) were made in each center according to their usual procedures.

### CXCR4 and CD26 surface expressions on lymphocytes subsets

The phenotypic characterization of lymphocyte subsets was performed using whole blood and standard immunofluorescence / flow cytometry technology. All the analyses were performed on EDTA-collected blood samples, in the department of immunology of the Saint Eloi University Hospital (Montpellier–France).

The following monoclonal antibodies were used for staining: CD3-Krome Orange, CD4-PC7, CD8-APC-Alexa Fluor 700, CD56-PC5.5, CD45RA-ECD, HLA-DR-Pacific Blue (Beckman-Coulter), CXCR4-APC and CD26-FITC (BD biosciences), IgG1-APC and IgG1-FITC (Beckman-Coulter).

Analyses were realized on whole blood. Erythrocyte lysis was realized with the Immunoprep solution on TQ-prep automat (Beckman-Coulter), after antibody fixation. Cells were analyzed by a Navios flow cytometer with the Kaluza software (Beckman Coulter).

The gating strategy is detailed in Additional file 2: Fig. S1. Total lymphocytes were identified based on morphological properties. T, T-helper, T-cytotoxic and natural killer (NK) lymphocytes were defined according to the following phenotypes: CD3+, CD3+ CD4+, CD3+ CD8+ and CD56+ CD3−. Naive T-helper and T-cytotoxic lymphocytes were defined as CD45RA+ CD4+ and CD45RA+ CD8+, respectively. CD4+ HLA-DR+ and CD8+ HLA-DR+ cells were considered as activated T-helper and T-cytotoxic lymphocytes.

The CXCR4 and CD26 expressions were measured on each subset by calculating the ratio of their MFI to the MFI of their isotype counterparts.

The B lymphocyte population was simply estimated by subtracting T and NK numbers from the total lymphocytes count.

### Statistical analysis

Clinical and biological characteristics are presented in tables. Immunological results are presented in bar charts with median and interquartile ranges or as individual values with Spearman r coefficient.

Considering the small size of the groups and the need to standardize the analysis of all the experiments, we only used non-parametric tests for quantitative variables. Comparisons between groups were made using the Mann–Whitney *U*-test (for two groups) or the Kruskal–Wallis test (for three groups or more). Correlations between variables were tested with the Spearman’s rank test. For categorical variables, the contingency tables were analyzed with the chi-squared test. All the univariate analyses were done with PRISM version 6.01 (GraphPad Software).

For the multivariate analysis, we used the website https://www.pvalue.io [49] to perform a linear regression. As the distribution of residuals did not follow a normal distribution, confidence intervals and p-values were calculated by bootstrap (1000 iterations).

A *p*-value < 0.05 was considered as statistically significant.

## Supplementary Information

**Additional file 1. Table SI:** Multivariable linear regression aimed at explaining the T-helper count as outcome variable in the 36 HHT patients. The explanatory variables tested were: CXCR4 MFI on T-helper lymphocytes, age and requirement of IV treatment for iron-deficiency anemia. Due to non-linearity in CXCR4 MFI on T-helper lymphocytes, this variable was divided in two classes (> 4.48 vs ≤ 4.48) for analysis.

**Additional file 2**. Gating strategy. (a) Total lymphocytes were selected in whole blood after erythrocyte lysis and staining, based on their morphological properties. Subpopulations of T lymphocytes and NK lymphocytes were determined based on the surface markers as follows: CD3+ for T cells, CD3+CD4+ for T-helper (CD45RA+ for naive, CD45RA- for memory and HLA-DR+ for activated), CD3+CD8+ for T-cytotoxic (CD45RA+ for naive, CD45RA- for memory and HLA-DR+ for activated), CD56+CD3- for NK. (b) CXCR4 and CD26 expression was assessed on T subpopulations by calculating the ratio of their mean fluorescence intensity (MFI) to the MFI of their isotype counterparts

## Data Availability

The datasets used and/or analyzed during the current study are available from the corresponding author on reasonable request.

## Abbreviations

HHT: Hereditary hemorrhagic telangiectasia
NK lymphocytes: Natural killer lymphocytes
HSI: History of severe infection
HC: Healthy control
AVM: Arteriovenous malformation
MNCs: Mononuclear cells
IV: Intravenous
MFI: Mean fluorescence intensity
