# Supplementary material for: Altered expressions of CXCR4 and CD26 on T-helper lymphocytes in hereditary hemorrhagic telangiectasia
Source: Orphanet J Rare Dis. 2021 Dec 14;16:511. doi: 10.1186/s13023-021-02139-y (PMC8670161; doi:10.1186/s13023-021-02139-y)
Supplement: Supplementary file 1 — Additional file 1. Table SI: Multivariable linear regression aimed at explaining the T-helper count as outcome variable in the 36 HHT patients. The explanatory variables tested were: CXCR4 MFI on T-helper lymphocytes, age and requirement of IV treatment for iron-deficiency anemia. Due to non-linearity in CXCR4 MFI on T-helper lymphocytes, this variable was divided in two classes (> 4.48 vs ≤ 4.48) for analysis. [file 13023_2021_2139_MOESM1_ESM.docx]

| **Adjusted correlation between T-helper count (cells/mm3) and:** | **Coefficients** | **p** |
| --- | --- | --- |
| CXCR4 MFI on T-helper lymphocytes (into classes: MFI>4.48 vs MFI≤4.48) | 142 [-55.5; 350] | 0.19 |
| Age (in year) | -11.5 [-22.4; -1.44] | **0.042** |
| Requirement of IV iron or blood transfusion (yes vs no) | -172 [-433; 52.4] | 0.13 |

Table SI: Multivariable linear regression aimed at explaining the T-helper count as outcome variable in the 36 HHT patients.

The explanatory variables tested were: CXCR4 MFI on T-helper lymphocytes, age and requirement of IV treatment for iron-deficiency anemia.

Due to non-linearity in CXCR4 MFI on T-helper lymphocytes, this variable was divided in two classes (>4.48 vs ≤4.48) for analysis.
