# Supplementary figures and images for: Altered expressions of CXCR4 and CD26 on T-helper lymphocytes in hereditary hemorrhagic telangiectasia
Source: Orphanet J Rare Dis. 2021 Dec 14;16:511. doi: 10.1186/s13023-021-02139-y (PMC8670161; doi:10.1186/s13023-021-02139-y)

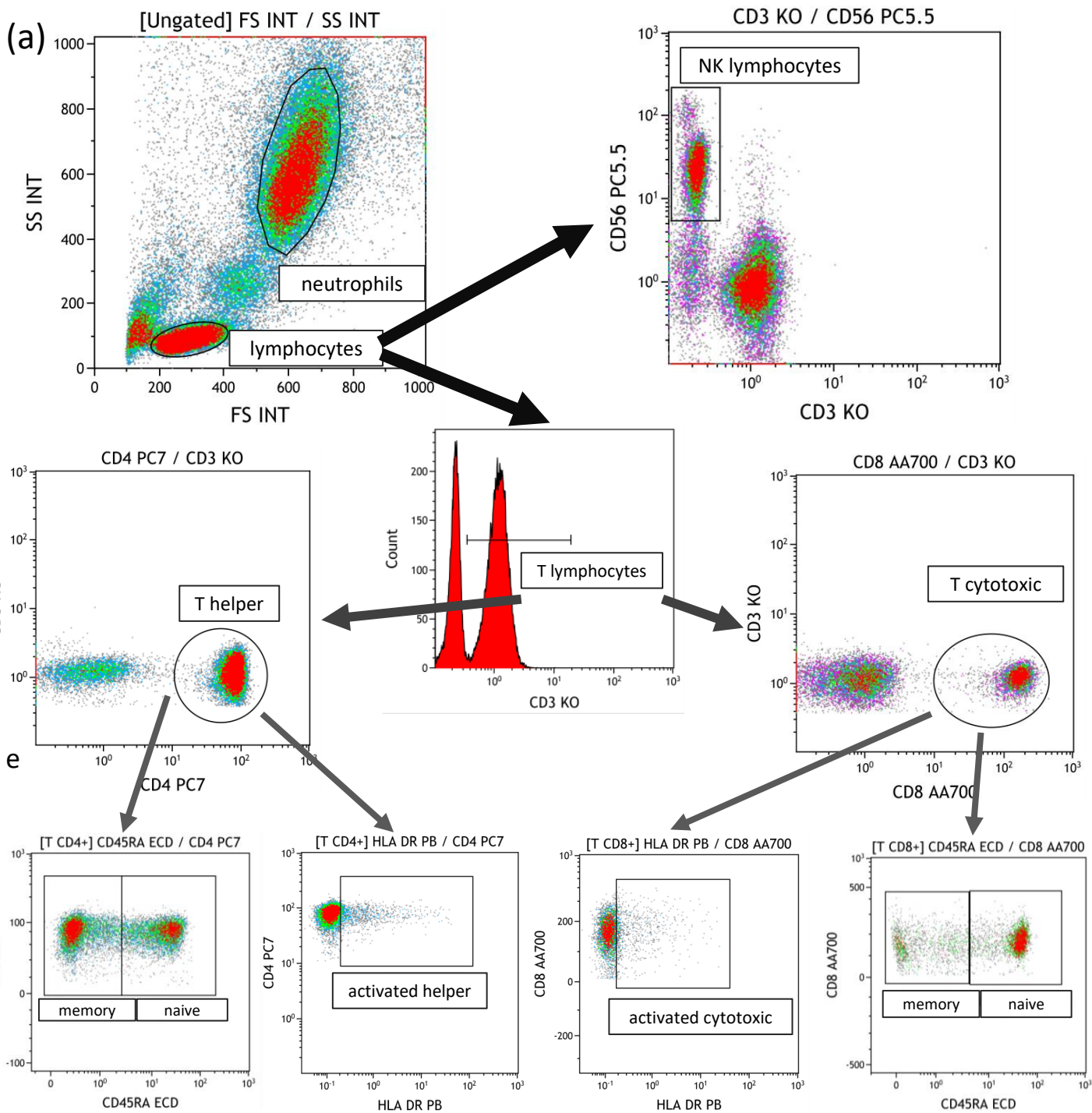

**(b)**

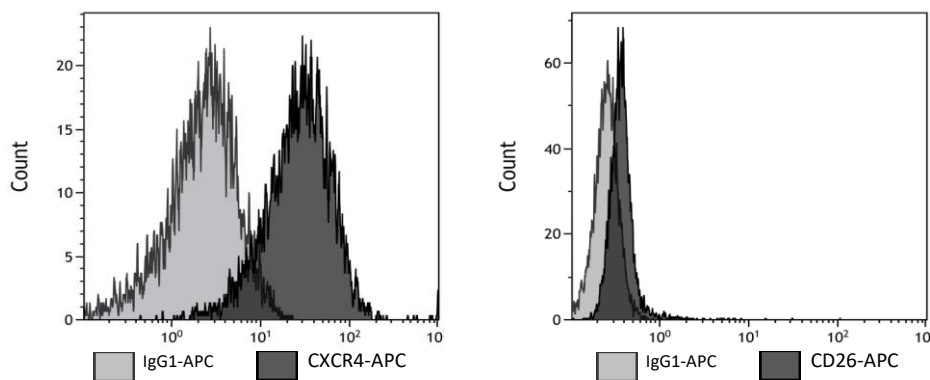

Supplement: Supplementary file 2 — Additional file 2. Gating strategy. (a) Total lymphocytes were selected in whole blood after erythrocyte lysis and staining, based on their morphological properties. Subpopulations of T lymphocytes and NK lymphocytes were determined based on the surface markers as follows: CD3+ for T cells, CD3+CD4+ for T-helper (CD45RA+ for naive, CD45RA- for memory and HLA-DR+ for activated), CD3+CD8+ for T-cytotoxic (CD45RA+ for naive, CD45RA- for memory and HLA-DR+ for activated), CD56+CD3- for NK. (b) CXCR4 and CD26 expression was assessed on T subpopulations by calculating the ratio of their mean fluorescence intensity (MFI) to the MFI of their isotype counterparts [file 13023_2021_2139_MOESM2_ESM.pdf]
